# Functional Phenotypes of Human Vγ9Vδ2 T Cells in Lymphoid Stress Surveillance

**DOI:** 10.3390/cells9030772

**Published:** 2020-03-22

**Authors:** Oliver Nussbaumer, Martin Thurnher

**Affiliations:** 1 GammaDelta Therapeutics Ltd., The Westworks, 195 Wood Lane, London W12 7FQ, UK; 2 Peter Gorer Department of Immunobiology, Kings College, London SE1 9RT, UK; 3 Immunotherapy Unit, Department of Urology, Medical University of Innsbruck, 6020 Innsbruck, Austria

**Keywords:** Vγ9Vδ2 T cells, butyrophilin, CD161, CD56, GPR56

## Abstract

Butyrophilin and butyrophilin-like proteins select γδ T cells and direct the migration of γδ T cell subsets to distinct anatomical sites. γδ T cells expressing Vδ2 paired with Vγ9 (Vγ9Vδ2 T cells) are the predominant γδ T cell type in human peripheral blood. Vγ9Vδ2 T cells, which cannot be studied easily in vivo because they do not exist in rodents, are often referred to as innate-like T cells. The genetically recombined γδ T cell receptor (TCR) that responds to isoprenoid-derived pyrophosphates (phosphoantigens) produced by infected and malignant cells in a butyrophilin-dependent manner qualifies them as therapeutically relevant components of the adaptive immune system. On the other hand, cell-surface proteins such as the C-type lectin CD161 mark a functional phenotype of Vγ9Vδ2 T cells that mediates TCR-independent innate-like responses. Moreover, CD56 (neural cell adhesion molecule, NCAM) and the G protein-coupled receptor GPR56 define Vγ9Vδ2 T cells with increased cytolytic potential and, like CD161, may also be expressed by dendritic cells, principally facilitating the generation of an innate-like immunological synapse. In this review, we summarise current knowledge of Vγ9Vδ2 T cell functional phenotypes that are critical to lymphoid stress surveillance.

## 1. Introduction

B cells, αβ and γδ T cells represent the three cell lineages that use unique heterodimeric antigen-binding surface receptors through the recombination of V(D)J genes [1]. Although the existence of γδ T cells was not anticipated [2], they turned out to be strikingly conserved within jawed vertebrates [3]. The ability of immune cells to generate specific antigen receptors by recombining germline-encoded gene segments, which are the hallmark of adaptive immunity, was acquired approximately 500 million years ago in jawed fish (gnathostomes, placoderms) [4]. Since then T cells expressing αβ T cell receptors to recognise peptide fragments of protein antigens presented on classical major histocompatibility complex (MHC) molecules have become the mainstream in T cell-mediated immunity. However, unconventional T cells expressing αβ or γδ T cell receptors that recognise non-peptide antigens in the context of unusual presenting molecules co-evolved. Despite their lower frequencies at least under resting conditions, these unconventional T cells appear to be equally important and add an extra line of defence.

Developing independently of MHC [5] in the thymus prior to αβ T cells [6], γδ T cells show unique patterns of selection and migration into specific anatomical sites based on their T cell receptor γ chain [7]. Most famously, this tissue-specific localisation is exemplified by mouse Vγ5 dendritic epidermal T cells (DETC), which get selected by the butyrophilin-like (btnl) molecule Skint-1 expressed on thymic epithelial cells and subsequently migrate to and remain next to differentiated keratinocytes, the only cell type expressing Skint-1 outside of the thymus [8]. The concept of btnl family member-mediated selection of γδ T cells with specific T cell receptor (TCR) heterodimers to tissues has since been extended to mouse Vγ7 γδ T cells localising to the intestine and forming the majority of intraepithelial lymphocytes by interacting with heterodimers of btnl1 and 6 [9]. Whilst humans do not have DETC, most likely due to mutations in the SKINT gene locus [10], conservation of this mechanism was shown through human Vγ4 γδ T cells, which localise to human gut epithelium, where preferentially expressed BTNL3/8 heterodimers induce γδ TCR down-regulation [9]. It has since been confirmed that BTNL3 directly binds to human Vγ4 TCRs via the germ line encoded complementarity determining region (CDR)2, which allows the γδ TCR to additionally bind to antigens via the somatically recombined CDR3 separating tissue selection from antigen recognition [11,12].

The examination of γδ T cell biology including tissue-specific localisation through distinct γδ TCRs has revealed fundamental contributions of these cells to tissue homeostasis. Many forms of microbial as well as nonmicrobial stress upregulate surface expression of ligands for γδ TCRs or for NKG2D, which are therefore collectively referred to as stress antigens [13]. By responding to these stress-related antigens, γδ T cells initiate in a nonclonal and MHC-independent manner the lymphoid stress-surveillance response, which differs fundamentally from the myeloid cell-induced adaptive immune response [13,14]. In addition to infection, γδ T cells respond to pre-malignant and malignant events through TCR-dependent metabolite detection or innate recognition of stress antigens via NKG2D [15] and natural cytotoxicity receptors [16].

γδ T cells also sustain tissue integrity by supporting repair processes. For instance, when skin is injured, DETC use their Vγ5 TCR to specifically respond to damaged keratinocytes and contribute to wound healing and re-epithelialisation [17,18]. This concept has been extended to other epithelial-rich tissues such as the gastrointestinal tract [19].

## 2. The Unique Biology of Vγ9Vδ2 γδ T Cells

Whilst Vγ5 DETC are absent in humans, Vγ9Vδ2 TCR-expressing γδ T cells are unique to primates and not found in rodents [20]. Expressing almost exclusively Vγ9JP1 rearranged γ chain TCRs paired to a Vδ2 chain, these cells dominate the repertoire of γδ T cells in the blood of healthy adults representing between 1–10% of all T cells. More or less, Vγ9Vδ2 T cells appear to be restricted to peripheral blood and thus represent another example of selective γδ T cell localisation.

Vγ9Vδ2 T cells predominantly respond to a phosphorylated metabolite of the non-mevalonate methylerythritol phosphate (MEP) pathway, named hydroxy-3-methyl-but-2-enyl-pyrophosphate (HMBPP) [21]. This allows Vγ9Vδ2 T cells to respond to a variety of microbial infections [22] and malaria [23], as the non-mevalonate pathway of isoprenoid biosynthesis is used in eubacteria, cyanobacteria and most protozoa. Vγ9Vδ2 T cells may thus dominate the T cell response to various pathogens making up to 60% and more of peripheral blood T cells during acute infection [24]. The MEP starts to overlap with the mevalonate pathway of isoprenoids used by most eukaryotes and some bacteria at the synthesis of isopentenyl pyrophosphate (IPP), which is also recognised by Vγ9Vδ2 T cells, although much less effectively, when compared to HMBPP [25]. Vγ9Vδ2 T cells also recognise dimethylallyl pyrophosphate (DMAPP), the isomer of IPP, as well as the downstream metabolites farnesyl pyrophosphate (FPP) and geranylgeranyl pyrophosphate (GGPP) [21,26], indicative of continuous pathway surveillance. As all these metabolites share two phosphate groups, which are essential for γδ T cell recognition, they are often referred to as phosphoantigens (pAgs). The ecto-ATPase CD39 has been shown to exhibit isoprenoid diphosphate phosphohydrolase activity and to inactivate such Vγ9Vδ2 T cell pAgs, thus regulating duration and magnitude of the Vγ9Vδ2 T cell response [27].

Vγ9Vδ2 T cells display considerable functional plasticity, combining adaptive and innate features including high cytotoxic potential normally associated with NK cells. The invariant Vγ9Vδ2 TCR effectively recognises various bacteria and parasites, whilst with lower affinity also binds mevalonate-derived metabolites accumulating in infected or hypermetabolic, transformed cells. In this sense, the adaptive Vγ9Vδ2 TCR, although indirectly through BTN3 molecules, serves a pattern-recognising purpose allowing for immediate, MHC-independent responses. Supplementary to their strong cytolytic potential, Vγ9Vδ2 T cells secrete various cytokines [24,28]. More surprisingly, Vγ9Vδ2 T cells may acquire regulatory capacity or may even act as professional antigen-presenting cells [29].

Although Vγ9Vδ2 T cells differ from Vδ1 T cells in terms of antigen recognition and tissue residence [9,30], including an abundance in blood, Vγ9Vδ2 T cells are also regulated by members of the BTN and BTNL family [31]. pAg recognition by Vγ9Vδ2 T cells requires heterodimers of BTN3A1 and 3A2 [32], which are widely expressed and not tissue-specific. Activation of Vγ9Vδ2 T cells through BTN3A1/3A2 though relies on the intracellular B30.2 domain of BTN3A1 to bind pAg [33]. pAg binding causes a conformational change in the B30.2 domain [34]. However, how intracellular pAg binding to the B30.2 domain translates into the activation of Vγ9Vδ2 is not yet fully understood, but it involves co-association of BTN2A1, which binds to a Vγ9 germ line encoded region leaving the hypervariable CDR3 region of the TCR uninvolved [35].

Intracellular sensing of danger-associated pAgs through BTN3A1 allows for the recognition of metabolically hyperactive cells by Vγ9Vδ2 T cells as in the case of viral infection [36] or malignant transformation [37,38]. This fact can be further exploited clinically by inhibiting FPP synthesis in tumour cells using nitrogen-containing bisphosphonates (N-BPs). N-BPs cause the intracellular accumulation of IPP via N-BP mediated mevalonate pathway inhibition, rendering N-BP treated tumour cells targets of Vγ9Vδ2 T cells [39,40]. Under these conditions, the ATP-binding cassette transporter A1 can also mediate the release of intracellular IPP from dendritic cells (DC) in cooperation with apolipoprotein A-I and BTN3A1 [41]. The resulting increase of extracellular IPP is sufficient to induce Vγ9Vδ2 T cell proliferation.

## 3. Vγ9Vδ2 T Cell Phenotypes

Consistent with their MHC class II-independent activation, Vγ9Vδ2 T cells lack CD4 expression but may express CD8 although the functional purpose of CD8 on Vδ2 T cells remains unclear. In accordance with their residence in peripheral blood, Vγ9Vδ2 T cells also lack C-C chemokine receptor type 2 (CCR2), which enables other leukocytes to extravasate from blood vessels. 

When activated through their TCR, Vγ9Vδ2 T cells show cytotoxic responses through the release of granzymes, perforin and IFN-γ. Although less versatile than helper subsets of CD4^+^ conventional αβ T cells (Th2 cells), Vγ9Vδ2 T cells also display some cytokine plasticity. Vγ9Vδ2 T cells have for instance been shown to produce the Th2 cytokine IL-4 [30], preferentially in response to stimulation with phytohemagglutinin (PHA) and *Mycobacterium tuberculosis* antigens [42]. Furthermore, Vγ9Vδ2 T cells cultured with the cytokines IL-1β, TGFβ, IL-6 and IL-23 differentiate into IL-17 producing cells [43]. Interestingly, whilst Th17 represents an established phenotype in mouse γδ T cells [44], IL-17 producing Vγ9Vδ2 T cells remain a rare observation in clinical settings [14].

Although Vγ9Vδ2 T cells perform innate-like responses, they may also generate long-lived memory populations [45] and therefore attempts have been made to define naive and memory subsets based on the αβ T cell markers, CD45RA and CD27 [46]. While αβ T cells predominantly depend on co-stimulation via CD28, CD70-CD27 costimulatory interactions support Vγ9Vδ2 T cell activation and provide important survival and proliferative signals [47]. Among Vγ9Vδ2 T cells expanded with N-BP and IL-2, effector/memory-like T cells (T_EMs_, CD45RA^−^CD27^−^) predominate [48]. Moreover, in patients with chronic lymphocytic leukaemia, poor proliferative response to zoledronate, which is the most potent N-BP available for clinical use, correlated with an even more pronounced bias toward T_EM_ and with terminal differentiation towards effector/memory T cells re-expressing CD45RA (T_EMRA_, CD45RA^+^CD27^−^) [49]. In addition, other CD markers have been described that define the distinct features of Vγ9Vδ2 T cells.

## 4. CD161 Marks a Functional Phenotype of γδ T Cells Mediating Innate-Like Responses

While Vγ9Vδ2 T cells express genetically recombined T cell receptors (TCRs), the hallmark of adaptive immunity, they can also respond in an unconventional, TCR-independent way, i.e., innate-like manner and participate in lymphoid stress surveillance for example through NKG2D [50]. Additionally, Vγ9Vδ2 T cells often express the C-type lectin CD161. The CD161 antigen, also known as natural killer cell-surface protein P1A (NKR-P1A) is a single-pass type II integral membrane protein expressed as a disulphide-linked homodimer of 80 kDa. CD161 represents the single human ortholog of the family of NKRP1 genes in rodents [51], and thus study of CD161-expressing lymphocytes is currently restricted to the human system. Likewise, Vγ9Vδ2 T cells are also absent in rodents suggesting a special relationship between this particular γδ T cell subset and CD161. However, CD161 is not restricted to γδ T cells but can also be expressed by subsets of CD4^+^ and CD8^+^ αβ T cell subsets as well as subpopulations of NK cells. Mucosal-associated invariant T (MAIT) cells are also characterised by high expression of CD161 [52]. MAIT cells display a semi-invariant TCR repertoire based on a limited selection of TCRα and TCRβ chains that restricts them to the MHC class Ib antigen-presenting molecule MR1 [53]. MAIT cell activation occurs when riboflavin precursors produced by a variety of bacteria are presented on an MR1. Hence, both Vγ9Vδ2 T cells and MAIT cells use semi-invariant TCRs that recognise non-peptide antigens in the context of unconventional presenting molecules. In the presence of a TCR signal, interaction of CD161 with lectin-like transcript 1 (LLT1) may enhance interferon (IFN)-γ production [54]. However, a common feature of these CD161^+^ innate-like T lymphocytes as well as NK cells is the ability to respond to the interleukin (IL) combination IL-12 plus IL-18 in the absence of αβ or γδ TCR engagement [52,55]. IFN-γ production in response to IL-12 plus IL-18 corresponded significantly to the levels of CD161, with the greatest responses seen in the CD161^high^ population of both, αβ and γδ T cells [52]. Gene expression analysis of sorted CD161^+^ and CD161^–^ T cells, including γδ T cells, revealed a conserved transcriptional signature consistent with the functional phenotype. The genes encoding the subunits of the IL-12 and the IL-18 receptor were conserved across CD161^+^ T cells of all lineages, constituting the ability of these cells to respond to IL-18 plus IL-12 by producing IFN-γ in a TCR-independent manner.

## 5. CD56^+^ γδ T Cells Participate in Lymphoid Stress Surveillance

IL-15 has been suggested to function as a danger signal during tissue stress surveillance [56]. In this concept, IL-15 contributes to tissue protection by promoting the elimination of infected or damaged cells. However, when IL-15 expression is chronically dysregulated, it can also promote T cell-mediated tissue destruction, leading to disorders such as coeliac disease and type 1 diabetes. γδ T cells have been identified as an early component of lymphoid stress surveillance in response to tissue perturbations caused by microbial and nonmicrobial cues [13]. IL-15 has been shown to convert circulating Vγ9Vδ2 T cell precursors into potent effector cells with the ability to rapidly produce large amounts of IFN-γ even in the absence of specific (exogenous) antigens [26]. Effector Vγ9Vδ2 T cells induced by IL-15 displayed increased levels of CD161 and in addition, they expressed the cytotoxic lymphocyte-associated proteins CD96, perforin and CD56. 

CD56, also known as a neural cell adhesion molecule (N-CAM or NCAM1), belongs to the immunoglobulin (Ig) superfamily of cell adhesion molecules, which are large cell-surface glycoproteins containing Ig-like and fibronectin type III-homologous (FnIII) domains [57]. Three major NCAM isoforms generated via alternative splicing comprise identical extracellular domains but differ in the mode of their association with the cell-surface membrane. The 180 kDa and 140 kDa isoforms are both transmembrane proteins. The 180 kDa NCAM isoform is only expressed by neurons. In contrast, the NCAM isoform with a molecular weight of 120 kDa is attached to the membrane via a glycosylphosphatidylinositol (GPI) anchor.

In hematopoietic tissues, CD56 is expressed on several lymphoid cell types, including NK cells as well as αβ and γδ T cells. In addition, CD56 has been detected on primary and leukaemic myeloid cells. Immunoprecipitation from NK cells using the anti-Leu-19 monoclonal antibody followed by enzymatic deglycosylation has been shown to result in a 137-kDa protein [58]. Moreover, the CD56 protein detected on hematopoietic cells was found to be resistant to removal by phosphatidylinositol phospholipase C, which releases GPI-anchored membrane proteins. Accordingly, the sequence of leukocyte-derived N-CAM cDNA was found to be essentially identical with N-CAM cDNA from human neuroblastoma cells encoding the 140-kDa isoform of N-CAM [59], altogether indicating that CD56 on leukocytes corresponds to the 140 kDa NCAM transmembrane protein. CD56/NCAM, which contains five Ig domains and two FnIII repeats in its extracellular domain, is heavily glycosylated. Essentially, it is the major carrier of polysialic acid, which can consist of up to 200 α2,8-linked sialic acid residues [57]. The large extracellular Ig domains of NCAM can bind to the same domains of these molecules at the cell-surface of the same (cis-interaction) or other cells (trans-interaction) to form so-called homophilic (self-binding) adhesive bonds. While homophilic adhesion may well contribute to the activation of CD56^+^ leukocytes, a role in the cytolytic interaction between CD56^+^ NK cells and CD56/NCAM-expressing tumour cell targets has previously been excluded [59].

## 6. CD56^+^ γδ T Cells Have a Distinct Surface Phenotype and Display Increased Cytolytic Potential

Effector Vγ9Vδ2 T cells differentiated with IL-15 express CD56 [26]. In response to stimulation with Vγ9Vδ2 T cell pAgs, these CD56^+^ effector Vγ9Vδ2 T cells upregulated surface expression of lysosomal-associated membrane protein 1 (LAMP-1, also known as CD107a) and exhibited strong cytotoxicity against MOLT-4 tumour cells in vitro. When Vγ9Vδ2 T cells were expanded from peripheral blood mononuclear cells of healthy donors by stimulating the cells with isopentenyl pyrophosphate (IPP) and IL-2, 30–70% of expanded Vγ9Vδ2 T cells expressed CD56 on their surface [60]. Interestingly, only CD56^+^ Vγ9Vδ2 T cells were capable of killing squamous cell carcinoma and other solid tumour cell lines. This effect was suggested to be mediated by the enhanced release of cytolytic granules, because CD56^+^ γδ T cells expressed higher levels of CD107a compared with their CD56^–^ counterparts following exposure to tumour cell lines. The lytic activity of CD56^+^ Vγ9Vδ2 T cells appeared to involve the perforin-granzyme pathway and was mainly Vγ9Vδ2 TCR/NKG2D dependent. Importantly, CD56-expressing Vγ9Vδ2 T cells were also found to be resistant to Fas ligand and chemically induced apoptosis [60].

Likewise, the cytolytic effector function of human circulating CD8^+^ αβ T cells has been demonstrated to closely correlate with CD56 surface expression [61]. CD56^+^CD8^+^ αβ T cells were shown to contain high amounts of perforin and granzyme B and to exhibit strong anti-CD3 mAb redirected cytotoxicity against Fcγ receptor-bearing P815 target cells. These findings, which indicate that CD56 marks γδ T cells and CD8^+^ αβ T cells with increased cytolytic potential, have been corroborated using a reverse approach. To distinguish CD56^+^ T cells from CD56^–^ T cells, Chan et al. performed an immunophenotyping array using 41 NK cell and T cell biomarkers related to activation, co-stimulation, inhibition, cytotoxicity, adhesion, and development [62]. Compared with CD56^–^ T cells, CD56^+^ T cells expressed higher levels of γδ TCR and CD8. Additional markers of cytotoxic cells such as CD16, NKG2A, NKG2D, CD122, DNAM-1, and granzyme B were also expressed at higher levels in CD56^+^ T cells. Collectively, these observations suggest that CD56 expression defines a functional phenotype that marks effector cells equipped with increased cytotoxicity and effector cytokine-producing capacity.

The differentiation of Vγ9Vδ2 into cytotoxic effector cells is promoted strongly by IL-15 and correlates with upregulated expression of CD56 [26]. Interestingly, when IL-15 is combined with TGFβ, Vγ9Vδ2 T cells become a major source of IL-9 [63], which similarly to IL-4, contributes to anti-worm immunity as well as atopic disease and has been implicated in the regulation of tumour immunity. Although TGFβ suppresses CD56 expression, a subpopulation of these Th9-type Vγ9Vδ2 T cells is still CD56^+^ [64]. A comparison of CD56^+^ and CD56^–^ Th9-type Vγ9Vδ2 with regard to cytolytic potential and IL-9 production has not been performed so far but would certainly be of interest.

## 7. CD56^+^ Antigen-Presenting Cells Activate CD56^+^ γδ T Cells

CD56 can also be expressed by myeloid cells. We and others have previously detected a CD14^+^ DC-like subset in human blood that expresses HLA-DR and CD56 [65,66]. Evidence has been obtained that cells with a similar phenotype can differentiate from CD14^+^ monocytes in response to GM-CSF and type 1 IFN [67]. In addition, CD56 is also expressed by some myeloid leukaemias [58].

Stimulation of the CD56^+^ subset of peripheral blood mononuclear cells (PBMCs) containing both, DC-like cells and abundant γδ T cells, with the potent N-BP zoledronate and IL-2 elicited rapid expansion of CD56^+^ Vγ9Vδ2 T cells as well as the production of γδ T cell effector cytokines (IFN-γ, TNF-α) and the monokine IL-1β [65]. The absence of IL-4, IL-17 and IL-10 in these cultures indicated a strong Th1 bias in the response of CD56^+^ Vγ9Vδ2 T cells, which strictly depended on CD56^+^ DC-like cells, since depletion of CD14^+^ cells prior to stimulation with zoledronate/IL-2 almost completely abolished cytokine production and dramatically impaired expansion of Vγ9Vδ2 T cells. This is particularly noteworthy as CD56 can facilitate homophilic interactions in cis and trans. Along the same line, CD56^bright^CD11c^+^ cells have been shown to play a key role in the IL-18–mediated proliferation of Vγ9Vδ2 T cells [68].

Interestingly, in the presence of IL-2 co-stimulation, CD56^+^ DC-like cells could also activate NK cells, when statins (mevastatin, simvastatin) were used instead of zoledronate [69]. Treatment with statin/IL-2 apparently induced inflammasome activation, since production of IL-1β and IL-18, which mediated NK cell activation in collaboration with IL-2, depended on caspase-1 activity. Caspase-1 proteolytically cleaves the proforms of IL-1β and IL-18 thus facilitating cytokine release [70]. Of note, zoledronate was also capable of inflammasome activation in the setting of CD56^+^ PBMCs. As a consequence, zoledronate not only activated CD56^+^ Vγ9Vδ2 T cells but also NK cells [71], a previously unappreciated effect of N-BPs.

## 8. CD56^+^ DC-Like Cells Have a Distinct Phenotype and Express CD161 and CD122

To distinguish CD56^+^CD14^+^DC-like cells from CD56^–^CD14^+^DC-like cells, we previously performed a microarray analysis using blood samples from four different donors (unpublished data, available upon request). The expression of NCAM1 (CD56), KLRB1 (CD161), IL2RB (CD122) and GPR56 was significantly enriched in CD56^+^ DC-like cells from all four donors. While the emergence of NCAM1/CD56 served as a useful internal control, the association of CD161 with CD56 was well in accordance with our finding that IL-15 induces the expression of both, CD161 and CD56 [26]. Interestingly, IL-15 is also produced by DCs in response to type 1 IFN [72], and DCs induced with IL-15 were shown to express CD56 [67,73]. Both, type I IFN-DCs and IL-15-DCs exhibited increased T-cell stimulatory potential and more surprisingly NK cell-like cytotoxic activity. Moreover, DCs generated with IL-15 also secrete IL-15, which promotes γδ T cell proliferation and IFN-γ production [74]. Expression of CD56 can also be upregulated by IL-2 [75], which may not be surprising as the receptors that mediate IL-2 and IL-15 signalling share the IL-2/IL-15 receptor β chain (CD122) and both signal through the common γ-chain (CD132). Importantly, CD122 was also overexpressed within the CD56^+^ DC subset. Altogether, the co-expression of CD161 and CD56 not only on Vγ9Vδ2 T cells but also on DC-like antigen-presenting cells suggested that CD161 and CD56 mark a functional phenotype of immune cells involved in innate-like responses characterised by cytotoxicity and Th1-type cytokine production, most likely facilitated through CD56^+^ cell-to-cell interactions and a positive feedback loop mediated by IL-15.

## 9. CD56^+^ DC-Like Cells Express the G Protein-Coupled Receptor GPR56

Intriguingly, GPR56 also emerged from our gene expression analysis as a cell-surface receptor that is co-expressed with CD56. GPR56 belongs to the adhesion class of G protein-coupled receptors (GPCRs), which comprises 33 members in humans with a broad cellular distribution in the developing embryo, the reproductive tracts, and the nervous as well as the immune system [76]. Adhesion-GPCRs (aGPCR) are defined by a large extracellular region linked to a seven-transmembrane-spanning domain (TM7) via a so-called stalk region containing a GPCR proteolytic site (GPS). The unusually large N-termini of aGPCRs often contain common structural domains, including epidermal growth factor (EGF)-like, thrombospondin repeats, leucine-rich repeats (LRRs), lectin-like, immunoglobulin (Ig) and cadherins. However, the GPR56 N-terminus contains none of these modules but does contain a characteristic GPS.

GPCRs can be activated by ligands or are transactivated by receptor tyrosine kinases [77]. In addition, GPCRs may acquire constitutive activity, for instance, at high receptor density [78]. Once activated, they couple to Gα (α_s_, α_i_, α_q_ und α_12/13_), Gβ, and Gγ subunits to promote numerous cellular responses [77]. GPCRs can couple to phospholipase C beta (PLCβ; via Gα_q_) or adenylyl cyclase (AC; via Gα_s_). Canonical downstream signalling includes cyclic adenosine monophosphate (cAMP) formation by AC resulting in the activation of protein kinase A (PKA) as well as PLCβ-catalysed production of second messengers, which mobilise calcium (inositol trisphosphate, IP_3_) and activate protein kinase C (diacylglycerol, DAG). In addition to these signalling events, Gα_q_-coupled receptors also stimulate the small GTPase Rho, which has a key role in cell migration through the stimulation of ROCK, and the expression of growth-promoting genes through the stimulation of mitogen-activated protein kinases (MAPKs) including ERK [77].

GPR56 has for instance been shown to couple to Gα_12/13_ and induce cell migration in a Rho-dependent manner [79]. In addition, GPR56 has been shown to specifically associate with Gα_q/11_ and Gβ subunits [80]. In this context, the tetraspanin CD81 was found to promote/stabilise the GPR56-Gα_q/11_ association [80]. Tetraspanins act as molecular scaffolds by forming complexes with other cell-surface proteins, including integrins, immunoglobulin superfamily proteins, proteoglycans, growth factor receptors and membrane-bound growth factors as well as other tetraspanins [80,81].

## 10. GPR56 Is Expressed by Cytotoxic Human Lymphocytes Including γδ T Cells

Among lymphocytes, expression of GPR56 has previously been shown to be restricted to cytotoxic cell populations such as NK cells and T cells including γδ T cells [82]. Of note, all these cell populations also express CD56 indicating that GPR56/CD56 co-expression occurs in myeloid DCs and T cells.

Although CD45RA expression is generally associated with naive T cells, a subset of effector/memory T cells re-expresses CD45RA (termed T_EMRA_) after antigenic stimulation and expands during infection with cytomegalovirus and dengue virus [83]. A subpopulation of T_EMRA_ has been shown to express GPR56, and GPR56^+^ T_EMRA_ cells displayed a transcriptional and proteomic program with cytotoxic features that are distinct from effector/memory T cells. Moreover, GPR56^+^ T_EMRA_ cells displayed higher levels of clonal expansion and contained the majority of virus-specific T_EMRA_ cells.

The tetraspanin CD81, which promotes/stabilises the GPR56-Gα_q/11_ association [80], was also found to play a role in immunological synapse formation and sustained signalling during T cell activation [84]. CD81 is important for the organisation of CD3 and ICAM-1 at the immunological synapse and for proper CD3-mediated intracellular signalling.

## 11. CD161, CD56 and GPR56 May Be Critical Components of an Innate-Like Immunological Synapse

**Figure 1:**
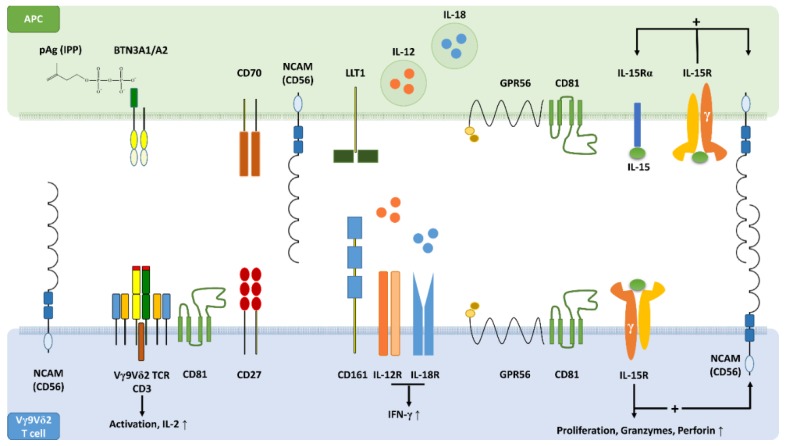
The concept of an innate-like immunological synapse. Schematic profile view showing key synaptic binding partners and signalling molecules that are involved in the activation of Vγ9Vδ2 T cells by antigen-presenting cells (APCs). Upon intracellular accumulation, pAgs such as isopentenyl pyrophosphate (IPP) bind to the B30.2 domain of butyrophilin (BTN)3A1 causing a conformational change of BTN3A1, which forms heterodimers with BTN3A2. Cognate interaction of Vγ9Vδ2 T cell receptors (TCRs) with BTN3A1/A2 initiates Vγ9Vδ2 T cell activation supported by CD70-CD27 costimulatory interactions. Homophilic adhesive bonds formed in trans between extracellular domains (EDs) of CD56 on APCs with EDs of Vγ9Vδ2 T cell CD56 may stabilise the immunological synapse. In addition, heterophilic binding of CD56 to other synaptic partners might be involved in synapse formation and maturation. In the presence of a TCR signal, interaction of CD161 with lectin-like transcript 1 (LLT1) may enhance interferon (IFN)-γ production. Alternatively to the pAg-dependent T cell activation, an innate-like immunological synapse may also facilitate the TCR-independent activation of Vγ9Vδ2 T cells. CD161^+^ Vγ9Vδ2 T cells can respond to interleukin (IL)-12 plus IL-18 derived from APCs by producing large amounts of IFN-γ. The stress-related cytokine IL-15 induces its own production in dendritic cells (DCs), the most professional APCs, as well as the expression of CD56 on DCs and Vγ9Vδ2 T cells. IL-15 also promotes proliferation and effector differentiation of Vγ9Vδ2 T cells, which express increased levels of granzymes and perforin. In DCs and Vγ9Vδ2 T cells, CD56 can be co-expressed with the IL-2/IL-15 receptor β chain (CD122) and with GPR56. GPR56 is a G protein-coupled receptor known to be associated with cytotoxic lymphocytes including Vγ9Vδ2 T cells. The tetraspanin CD81 supports GPR56 signalling and participates in immunological synapse formation. GPR56 may define a subset of terminally differentiated effector T cells with cytotoxic features and possibly anti-viral activity.

CD161, CD56 and GPR56 define a functional phenotype of Vγ9Vδ2 T cells and can also be expressed by myeloid DCs [26,82]. CD56 is identical to neural cell adhesion molecule (NCAM), which regulates synapse maturation in the nervous system through homophilic and heterophilic interactions with recognition molecules [57]. CD56 may therefore also be a critical component of a distinct immunological synapse that promotes innate-like responses (Figure 1). CD161 may co-stimulate pAg-dependent Vγ9Vδ2 T cell activation on the one hand, and on the other play a central role in TCR-independent Vγ9Vδ2 T cell responses. Although still poorly defined, GPR56 appears to participate in the signalling processes required for cytotoxic responses [82,83]. IL-15 is expected to play a key role in such an innate-like immunological synapse (Figure 1). By stimulating its own production [74], IL-15 may generate a feed-forward loop promoting the expression of CD161, CD56 [26] and possibly also that of GPR56.

## 12. Conclusions and Outlook

Vγ9Vδ2 T cells contribute to lymphoid stress surveillance by recognising cells that accumulate either self-pAgs as a consequence of metabolic dysregulation or microbial pAgs during infection. Even in the absence of pAgs, Vγ9Vδ2 T cells with a distinct functional phenotype defined by CD161 expression can respond to DC-derived IL-12 and IL-18 by producing large amounts of IFN-γ. Interactions between Vγ9Vδ2 T cells and DCs are driven by IL-15, a stress-related cytokine that serves as an alarmin and upregulates CD161 and CD56 on either cell type. IL-15 also stimulates its own production and may, therefore, be central in the formation of a potential innate-like immunological synapse that may especially promote lymphoid stress surveillance. Intriguingly, the adhesion class GPCR, GPR56, is associated with CD56 and CD161 expression on both, Vγ9Vδ2 T cells and DCs. Although little is known about GPR56, it appears to be associated with cytotoxic potential. Moreover, GPR56 is expressed by terminally differentiated effector αβ T cells that may be critical for the control of viral disease [83]. Since Vγ9Vδ2 T cells may also participate in viral surveillance [85], the role of GPR56 in Vγ9Vδ2 T cell biology certainly deserves further investigation.

As innate-like T cells that combine adaptive properties of conventional αβ T cells and innate features of NK cells, γδ T cells are highly attractive candidates for cell-based immunotherapy of infectious and malignant disease [86]. Recently, attempts have been made to equip these already versatile innate-like T cells with additional functions. Using genetic manipulation, chimeric antigen receptors (CARs) or αβ T cell-derived TCRs have been transferred to γδ T cells in order to combine tissue-resident biology and innate target recognition with antigen-specific activation and selection [87]. Along the same line, Vγ9Vδ2 T cells have been genetically engineered to acquire chemotherapy resistance enabling the combined administration of chemo- and immunotherapy [88]. This is of particular interest because Vγ9Vδ2 T cell recognise NKG2D ligands, which are upregulated on tumour cells in response to chemotherapy. In addition to genetic manipulation, novel bispecific antibodies simultaneously binding Vγ9 on γδ T cells and Her2/neu (ERBB2) expressed by tumour cells have been used to increase Vγ9Vδ2 T cell antitumour cytotoxicity [89]. In its various forms [87,90], Vγ9Vδ2 T cell-based immunotherapy may be optimised by the Vγ9Vδ2 T cell subset selection. As outlined in the present review, Vγ9Vδ2 T cells expressing CD161, CD56 and GPR56 might be preferred cell populations for immunotherapy purposes.
